# COVID‐19 in pregnancy—what study designs can we use to assess the risk of congenital anomalies in relation to COVID‐19 disease, treatment and vaccination?

**DOI:** 10.1111/ppe.12840

**Published:** 2022-03-02

**Authors:** Helen Dolk, Christine Damase‐Michel, Joan K Morris, Maria Loane

**Affiliations:** ^1^ Ulster University United Kingdom; ^2^ INSERM Centre Hospitalier Universitaire de Toulouse France; ^3^ St George’s University of London United Kingdom

**Keywords:** congenital anomalies, *COVID‐19*, healthcare databases, pregnancy cohorts, registries, study design

## Abstract

**Background:**

The COVID‐19 pandemic has accelerated pregnancy outcome research, but little attention has been given specifically to the risk of congenital anomalies (CA) and first trimester exposures.

**Objectives:**

We reviewed the main data sources and study designs used internationally, particularly in Europe, for CA research, and their strengths and limitations for investigating COVID‐19 disease, medications and vaccines.

**Population:**

We classify research designs based on four data sources: a) spontaneous adverse event reporting, where study subjects are positive for both exposure and outcome, b) pregnancy exposure registries, where study subjects are positive for exposure, c) congenital anomaly registries, where study subjects are positive for outcome and d) population healthcare data where the entire population of births is included, irrespective of exposure and outcome.

**Study Design:**

Each data source allows different study designs, including case series, exposed pregnancy cohorts (with external comparator), ecological studies, case‐control studies and population cohort studies (with internal comparator).

**Methods:**

The quality of data sources for CA studies is reviewed in relation to criteria including diagnostic accuracy of CA data, size of study population, inclusion of terminations of pregnancy for foetal anomaly, inclusion of first trimester COVID‐19‐related exposures and use of an internal comparator group. Multinational collaboration models are reviewed.

**Results:**

Pregnancy exposure registries have been the main design for COVID‐19 pregnancy studies, but lack detail regarding first trimester exposures relevant to CA, or a suitable comparator group. CA registries present opportunities for improving diagnostic accuracy in COVID‐19 research, especially when linked to other data sources. Availability of inpatient hospital medication use in population healthcare data is limited. More use of ongoing mother‐baby linkage systems would improve research efficiency. Multinational collaboration delivers statistical power.

**Conclusions:**

Challenges and opportunities exist to improve research on CA in relation to the COVID‐19 pandemic and future pandemics.

**Synopsis**

**Study Question**

We reviewed the main data sources and study designs used internationally, particularly in Europe, for CA research, to determine their strengths and limitations for investigating COVID‐19 disease, medications and vaccines.

**What is already known**

Most pregnancy research related to the COVID‐19 pandemic, whether the disease, its treatment, or vaccines, has concerned second and third trimester exposures.

**What this study adds**

This study investigates how we can generate more high‐quality evidence about the adverse effects of periconceptional and first trimester exposures, specifically in relation to congenital anomalies.

## 1 INTRODUCTION

The COVID‐19 pandemic has accelerated timelines for health research to generate real‐world evidence. One of the areas in which this is needed is pregnancy outcome research and specifically the risk of congenital anomalies (CA). SARS‐CoV‐2 infection and associated morbidity, its treatment and the vaccines all have theoretical potential to adversely affect foetal development. The COVID‐19 pandemic could also have indirect effects on CA risk through factors such as altered periconceptional care particularly for chronic diseases (such as diabetes), stress, nutrition or other exposures.

Risks during pregnancy, whether to the pregnant woman or to the unborn baby, are sensitive to the timing of the exposure. Currently, the majority of evidence regarding COVID‐19 during pregnancy concerns second and particularly third trimester exposures, when pregnancy carries a higher risk of severe disease and need for intensive care.^1^, ^2^, ^3^ CA relate mainly to first trimester and periconceptional exposures, and there is, therefore, a delay before the outcome of pregnancy is known. During the first pandemic wave, most pregnant women who were tested were in their second or third trimesters^1^, ^2^ so the number of women with confirmed COVID‐19 infection in their first trimester and known outcome from that pandemic period remains low. During the second wave in Europe, more non‐hospitalised cases were confirmed by SARS‐CoV‐2 tests in pregnant women, and these women are delivering their babies in mid‐2021. Unfortunately, many extant studies of pregnancy do not present trimester‐specific information.^4^ CA are often not reported at all, or are considered together as one heterogeneous group, thus missing the opportunity to assess the potential impact of infections or medications on specific types of congenital anomalies.^5^

In this paper, we review the main data sources and study designs being used internationally and particularly in Europe for CA research, and their strengths and limitations for COVID‐19 research. We begin by briefly reviewing the current evidence gaps for COVID‐19 in pregnancy and CA risk.

## 2 COVID‐19 IN PREGNANCY AND RISK OF CONGENITAL ANOMALY: THE EVIDENCE SO FAR AND RESEARCH GAPS

### 2.1 COVID‐19 and congenital anomaly risk

COVID‐19 is a respiratory disease caused by the SARS‐CoV‐2 virus, a virus which binds to human angiotensin‐converting enzyme2 (ACE2) receptors. The most common symptoms of COVID‐19 in pregnant women are similar to those in the general population,^2^, ^6^, ^7^, ^8^, ^9^ however, pregnant women are at greater risk of severe disease, and are more likely to need intensive care or mechanical ventilation than non‐pregnant women.^2^, ^3^, ^4^, ^10^, ^11^, ^12^ Pregnant women represent a vulnerable group to the effects of respiratory disease, including influenza, due to immunological changes and physiological adaptive changes during pregnancy, for example diaphragm elevation, increased oxygen consumption. There is also evidence of thromboembolic maternal complications, especially in the final stages of pregnancy.^10^, ^11^ The presence of pre‐existing medical conditions such as obesity, asthma, hypertension and diabetes is strongly associated with an increased risk of severe COVID‐19 disease,^2^, ^4^, ^14^, ^15^ and CA research will need to distinguish the effects of these underlying diseases on CA risk from COVID‐19. In general, however, the risk of severe maternal COVID‐19 disease is greater in the second and third trimesters, after the main period of risk for CA.

Other coronaviruses, such as SARS‐CoV and MERS‐CoV, have been reported to cause severe adverse pregnancy outcomes including miscarriage, premature delivery, intrauterine growth retardation and maternal death.^16^ There have not, however, been reports of elevated CA risk following first trimester exposure in relation to other coronaviruses, although cohorts of infected pregnancies have been small and therefore specific risks may have been missed. In general, maternal infection is a well‐established cause of congenital anomalies, each infection with a specific syndromic pattern, such as congenital zika syndrome^17^ and congenital rubella.^18^ There is evidence that influenza is teratogenic^19^ although the evidence is not as consistent and there may be cofactors which influence risk. Regardless of the infective agent, there is considerable evidence that hyperthermia and fever can be teratogenic^19^ and therefore fever associated with COVID‐19 in the first trimester could be teratogenic.

Vertical transmission of SARS‐CoV‐2 has been documented,^20^, ^21^, ^22^, ^23^, ^24^ but is thought to occur at low rates. Thromboembolic complication associated with the SARS‐CoV‐2 infection may lead to foetal vascular malperfusion or foetal vascular thrombosis.^25^, ^26^, ^27^ A systematic review of evidence up to October 2020 indicated increased risk of preterm birth, stillbirth and admission to neonatal intensive care.^4^ Evidence relating to CA and first trimester infection specifically, has not been systematically reviewed, and there is a need for studies to present results by trimester of infection to facilitate this. A case‐series of nine pregnant women who had confirmed maternal SARS‐CoV‐2 infections in the first trimester of pregnancy, found that one child was born with a severe eye anomaly (unilateral microphthalmia, optic nerve hypoplasia, and congenital retinopathy).^28^ The potential for more case reports where exposure is so common emphasises the need for controlled epidemiological studies to assess risk.

### 2.2 COVID‐19 medications and congenital anomaly risk

Prescribing of medications to pregnant women requires the beneficial effect on the mother to be weighed up against its potential adverse effects, not only for her but also for her unborn child. The risks to be considered include not only those from exposure to the medicine when used, but also the risk of untreated disease for the woman and the unborn child when no medicine is used. Exposures early in pregnancy, relevant to CA, may occur before the woman knows she is pregnant. Moreover as medication exposure represents the time period when a medication and its metabolites are present in the pregnant woman's body, medication use before the start of pregnancy has to be considered depending on each drug's pharmacokinetic characteristics (eg the terminal elimination half‐life of hydroxychloroquine is more than 40 days).^29^ Pregnant women are usually excluded from clinical trials. Safety information regarding pregnancy exposure must, therefore, be obtained mainly from postmarketing surveillance which has historically been a slow and inadequate process leaving huge evidence gaps.^5^, ^30^

Medications used to treat COVID‐19 (Supplementary Appendix 1)^31^ vary according to the severity of the disease.^32^, ^33^ The therapeutic strategies are likely to vary between countries. For instance, in the United States, studies of pregnant women early in the pandemic indicated frequent exposure (usually after the first trimester) to hydroxychloroquine, Remdesivir and azithromycin.^2^, ^14^ The WHO Solidarity trial recommended dropping Remdesivir, hydroxychloroquine, lopinavir and interferon regimens as they had little or no effect on hospitalised COVID‐19,^34^ but pregnancy exposures are likely to have occurred to these drugs. Their placental transfer and toxicity data to date have been reviewed elsewhere.^31^, ^35^, ^36^ Additional concerns have been raised regarding risk to the fetus of hydroxychloroquine,^37^ azithromycin^38^ and anticlotting agents.^39^.

**TABLE 1:** Types of pregnancy exposure registry, and their characteristics

Type of pregnancy exposure registry	Characteristics	Examples (general and COVID)
Clinically led disease‐based pregnancy exposure registries	Recruit pregnant women diagnosed with a specific disease, and can compare outcomes according to the medication or treatment used. Can have high recruitment and retention^1^, ^46^. Recruitment may be via disease specialists looking after high risk pregnant women (e.g., neurologists^46^), or via obstetricians and maternity units.^1^, ^48^, ^50^, ^51^, ^52^ Recruitment may involve all eligible clinicians^1^ and be nearly population‐based, or only clinicians who elect to participate^46^, ^50^, ^51^. Some systems have been repurposed for COVID‐19^1^, ^50^, ^51^	EURAP (Epilepsy)^46^ Obstetric Surveillance Systems^1^, ^53^, ^54^ INTERCOVID based on Intergrowth Study^51^ COVIPreg based on Zika pregnancy cohort^50^
Teratogen Information Service cohorts.	Teratogen Information Service cohorts are opportunistic cohorts where women who contact the service about a pregnancy exposure, either themselves or via their health professional are enrolled and followed up to ascertain pregnancy outcome. Recruitment may be enhanced for specific studies.^49^ The MothertoBaby studies of the OTIS network recruit pregnant women with exposure to medications of interest, with the disease indication, and without these exposures as comparator and are studying COVID‐19 infection, medication and vaccine.	ENTIS^55^ (www.entis‐org.eu) OTIS/ MothertoBaby^49^ (www.mothertobaby.org)
Industry pregnancy registries.	Industry pregnancy registries are instituted to provide safety data for single medicinal products that may be used by pregnant women. The record of such registries in terms of recruitment of exposed pregnant women, completeness of follow up (attrition), lack of comparator data, and quality of data about congenital anomalies, has been poor.^47^ However, for new COVID‐19 medications, industry pregnancy registries are likely to become an important component of the safety monitoring system.	List of Pregnancy Exposure Registries | FDA https://www.fda.gov/science‐research/womens‐health‐research/list‐pregnancy‐exposure‐registries
Direct to mother cohorts.	“Direct to Mother” approaches bypass the healthcare system to recruit pregnant women directly.^56^ IRCEP advertises for participants via social media and online parenting forums,^57^ following up monthly via an app until 90 days after delivery. PRIORITY uses a partially direct to mother approach, but also recruits pregnant women with confirmed or suspected COVID‐19 via healthcare providers^58^ and confirms birth defects with electronic medical records from the hospital of birth.^58^	The US‐based International Registry of Coronavirus in Pregnancy (IRCEP) uses the pre‐existing PREGISTRY platform.^57^ The US PRIORITY Study (Pregnancy Coronavirus Outcomes Registry)^58^
Vaccine safety pregnancy registries recruiting via vaccination centres	Recruitment of pregnant women via vaccination centres rather than via maternity units.	vSafe^43^ COVACPREG^59^

### 2.3 COVID‐19 vaccine and congenital anomaly risk

Since pregnant women were excluded from initial clinical trials of COVID‐19 vaccines, and vaccines have only been available since late 2020, evidence regarding safety during the first trimester of pregnancy is at an early stage. Two population‐based case‐control studies of spontaneous abortions did not find any excess risk in COVID‐19 vaccinated women.^40^, ^41^ A rapid review of studies assessing vaccines and vaccine components which relate to COVID‐19 vaccines in pregnant women^42^ found no indication of increased CA risk, but based on low numbers of exposed women evaluated. No comparative studies are available to date on risk of CA following first trimester vaccination. The V safe pregnancy registry has so far reported mainly on third trimester vaccinations.^43^ Evidence from first trimester exposures, including exposures before the pregnancy was recognised, will only be available in late 2021.

## 3 TYPES OF DATA AND OPTIONS FOR STUDY DESIGN

**FIGURE 1:**
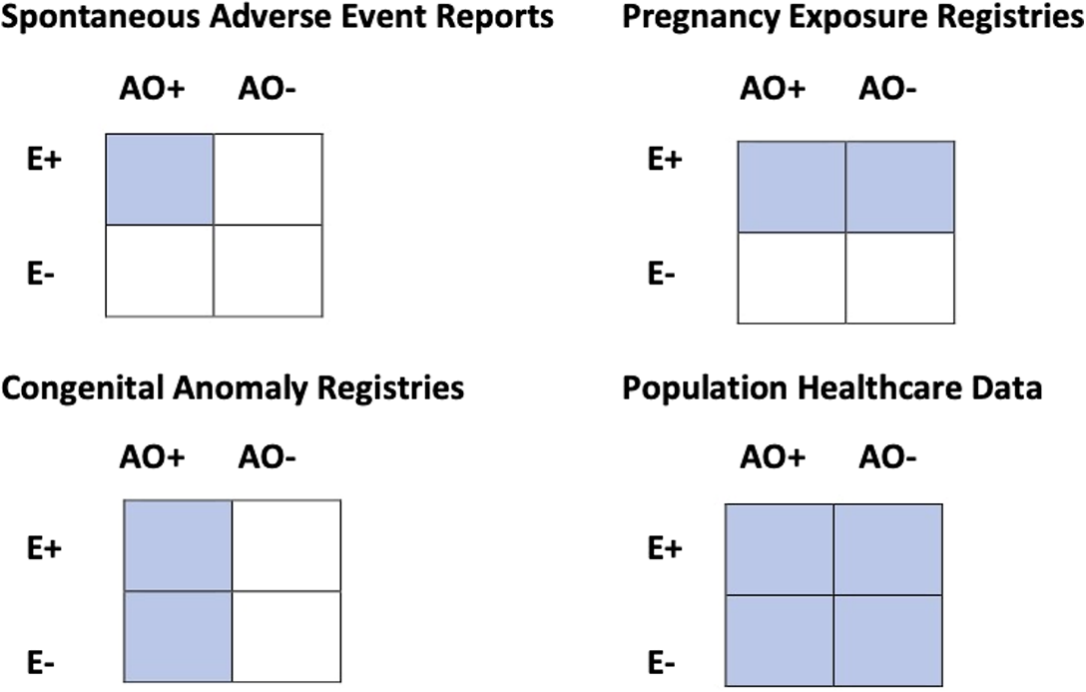
Types of data used in pregnancy pharmacovigilance with shaded area according to whether study subject selection is according to exposure to maternal medication/disease/vaccination, or according to presence of adverse pregnancy outcome (AO, Adverse Outcome; E, maternal exposure)

There are four types of data (Figure 1) which can be used for pregnancy pharmacovigilance and disease‐related risk studies. These can support a variety of possible study designs.

### 3.1 Case series and Spontaneous Adverse Event Reports

Spontaneous Adverse Event Reports (SAER) are alerts sent by doctors or patients to industry or regulators to report a suspected adverse effect of a medicine or vaccine. In the context of pregnancy, these may also report cases of adverse pregnancy outcome with maternal medication/vaccine exposure. These could be examined as case report series for evidence that a specific type of CA is being disproportionately reported, however, they may be poorly specified, particularly in terms of the exact nature of any congenital anomaly and the exact timing of the exposure during pregnancy. Reporting depends on suspicion of causality, and may favour known teratogens. There are no comparator data other than historical data, and no denominator data regarding the total number of exposures. Nevertheless, SAER data have identified important safety signals in the past.^44^ In relation to COVID‐19 medication and vaccine, while SAERs cannot be relied upon, they are an important adjunct in monitoring novel treatments or vaccines.^43^

### 3.2 Pregnancy exposure registries and disease cohorts

‘Pregnancy exposure registries’,^45^ sometimes called ‘pregnancy registries’, are prospectively followed cohorts of pregnant women exposed to the medication(s) or disease of interest. They typically recruit subjects prospectively (before the outcome of pregnancy is known) to avoid bias due to selective inclusion of exposed pregnancies with an adverse outcome. Advantages to prospective primary data collection include the potential for rapid data availability (within the constraints of delay from exposure to pregnancy outcome), use of standard definitions and detailed exposure data (eg COVID‐19 symptoms), and potential to set up these cohorts/registries in all countries which may lack other sources of data. However, they have tended to suffer from low recruitment and small sample sizes and, therefore, have mainly been useful as initial indicators of high overall CA risk, rather than investigating the risk of specific CA.^5^ Of the five types of pregnancy registry (Table 1), those led by clinicians in a specialty area (e.g. neurology^46^) who need safety information to guide treatment decisions have been the most successful at recruiting large numbers of women. Industry registries have a particularly poor record of recruitment and follow‐up.^47^ The other main disadvantage of pregnancy registries has been lack of an internal (unexposed) comparator

The COVID‐19 pregnancy exposure registries have some limitations in relation to CA research. First, many record only COVID‐19 infection which led to hospitalisation, usually in later trimesters, or at the time of delivery when routine testing is done. Non‐hospitalised COVID‐19 in the first trimester (and its treatment) is underrepresented. Moreover, during the first pandemic wave, non‐hospitalised cases were rarely confirmed by tests. Second, trimester of infection is frequently not reported,^4^ so that reports of CA among infected pregnancies are diluted by data from pregnancies infected after the vulnerable period. Third, few have an internal comparator of uninfected women. For CA, this is a particularly problematic as CA reporting requires standardisation. Some registries use historical data, such as the UK Obstetric Surveillance System,^1^ and the French COROPREG,^48^ others have sought to include women without infection in their protocol.^49^ Other common limitations include lack of very early exposures before the pregnancy has been recognised (but relevant to CA risk), exclusion of TOPFA, lack of follow up after birth^50^ for major congenital anomalies diagnosed later, such as heart defects, and lack of standardised reporting protocols for CA.

### 3.3 Congenital anomaly registry‐based designs

In Europe, population‐based CA registries (Figure 1) cover nearly one third of the European birth population, and many of these CA registries contribute to the EUROmediCAT Central Database^60^ and national data linkage resources (Supplementary Appendix 2).^60^ The strengths of congenital anomaly registries are that they provide good diagnostic data on specific congenital anomalies; provide more complete ascertainment of CA; cover a very large population to investigate rare exposures, rare CA or moderate risks of more common CA; and include TOPFA and CA diagnosed after the neonatal period.

Congenital anomaly registries increasingly use electronic healthcare data for case ascertainment, but the work of the registry is to make sure that the diagnosis in each case is validated, using multiple sources of information and expertise in medical genetics, paediatrics and foetal medicine. Diagnostic accuracy is important so that CA can be analysed as specific predefined subgroups,^61^ since an increase in risk in specific subgroups can be missed if all CA are considered together,^5^ or major and minor anomalies are combined. TOPFA comprises a high proportion of severe congenital anomalies in Europe,^62^ and no study of risk of COVID‐19 or its treatment can be valid without their inclusion. This is particularly important in relation to COVID‐19, since delivery of antenatal care changed during the pandemic, and the frequency of TOPFA may vary in relation to COVID‐19 disease or medication characteristics, and thus, their exclusion can bias estimates of relative risk. CA surveillance networks have provided a number of resources which can be useful for congenital anomaly studies in relation to COVID‐19, whether or not they use congenital anomaly registry data (Supplementary Appendix 3).

Congenital anomaly registries obtain exposure data mainly from medical records (eg obstetric records) prospectively recorded during pregnancy, supplemented by other sources including interview for some registries.^63^, ^64^ While information on COVID‐19 test results, medications and vaccines can be obtained from maternity records (and specific fields have been added in EUROCAT data collection), this information may not be recorded when not directly relevant to the care episode. In eight European countries (Supplementary Appendix 2), it is now also possible to link congenital anomaly registries to prescription data, which considerably enhances the exposure information available.^64^ Prescription data for medications given in hospital settings, however, are generally not centralised in population databases, and hospital prescriptions for inpatient treatment therefore need to be separately ascertained.^63^ In the COVID‐19 context, linkage to databases of COVID‐19 test results and COVID‐19 vaccination records is also highly relevant where available. A limitation is that not all registries have a unique identification number provided at birth which can be used to link to other electronic data sources and, in particular, linkage may not be possible for TOPFA. The COVID‐19 pandemic is encouraging the development of linkages between congenital anomaly registries and databases of maternal exposure, but this is still not possible in many European countries.

#### 3.3.1 Ecological (surveillance) studies

Ongoing surveillance of congenital anomaly rates is a core activity for congenital anomaly registries and is likely to be useful for the COVID‐19 situation, particularly in the early stages of the pandemic when there was little population testing and, thus, limited ability to determine COVID‐19 infection status of pregnant women on an individual basis. Ecological studies have been used in relation to pregnancy exposures during influenza outbreaks. For example, an ecological study was performed to determine whether congenital anomaly prevalence in Europe was related to the intensity of population infection during the 2009 H1N1 pandemic using weekly infection data relating to the vulnerable period in early pregnancy.^65^ Ecological studies can be used to compare the incidence of adverse outcomes before and after vaccine introduction, but this design has been little used in studies of vaccines in pregnancy. ^42^, ^65^

Ecological studies describe the overall effects of the pandemic which also include altered healthcare, stress, nutrition and other factors, and changes in reporting of CA must also be considered.^66^ It may be difficult to distinguish the effect of COVID‐19 infection (and comorbidities) from treatment. However, international studies can be useful in this regard as differences in treatment between countries provide a natural experiment. Another consideration is differentiating the societal effects of the pandemic from the effects of infection or its treatment; for example, studies in some populations have found preterm births to have decreased during COVID‐19 lockdowns,^67^ at the same time as pregnant women with COVID‐19 disease have been at increased risk.^4^

#### 3.3.2 Case‐control studies, with non‐malformed or malformed controls

Population‐based congenital anomaly registries can also provide a basis for case‐control studies, using malformed controls or additionally collecting data on non‐malformed controls. The population‐based nature of CA registries ensures that referral biases found in hospital‐based studies (eg due to referral of high risk pregnancies or those with positive prenatal screening findings, to tertiary hospitals^68^) are minimised.

Case‐control surveillance is a system whereby for each malformed case born, a number (eg two) of non‐malformed controls are chosen. This approach has been used on an ongoing basis by ECLAMC, the Latin American hospital‐based congenital anomaly surveillance system, which collects data on risk factors by interviewing mothers before discharge from hospital.^68^ It is also used in the United States by the population‐based National Birth Defects Prevention Study and its successor BDSteps^69^ where mothers are interviewed by telephone during the months or years after birth and by the Slone Birth Defect Study,^71^ which focuses on medication exposures. Retrospective interviews can introduce recall bias if mothers of cases recall their exposure status differently than a mother of a control baby. Questions about COVID‐19 can be introduced, and verification or linkages with records of prescription, test and vaccination are also possible if the appropriate consent procedures are in place.

In Europe, the majority of congenital anomaly registries do not collect data on non‐malformed controls. Instead, case‐control studies can be performed with malformed controls, where the controls either have genetic anomalies (which cannot be due to medication exposure or infection during pregnancy) or anomalies not associated with the primary hypothesis of interest.^71^ The advantage of this design is that exposure data are collected in the same way for cases and controls, including any data that may be affected by the possibility of maternal recall bias.^71^ The disadvantages are the possibility of ‘teratogen non‐specificity bias’, where the controls may include CA associated with the exposure in question, thus diluting risk estimates,^72^ and the inability to produce an overall estimate of CA risk related to the exposure. Such studies examine specificity of effect. Signal testing (signal evaluation) studies, with a hypothesis based on prior evidence, test an exposure's association with a signal CA by comparing it to control CA which have not been associated with the exposure in question. Signal detection studies, without prior hypothesis, look for an overrepresentation of specific CA‐medication combinations and use a variety of methods for disproportionality analysis and for adjusting the false positive rate (e.g., by use of the False Discovery Rate).^73^ Such studies could detect new signals due to the introduction of new medications or medication combinations to treat COVID‐19

### 3.4 Population healthcare data or birth cohorts

The fourth type of data relates to all births in the population (unselected by exposure or outcome, Figure 1), whether this is obtained from secondary use of existing data sources, particularly electronic healthcare databases (which can be linked to congenital anomaly registers), or by primary data collection involving recruitment of pregnant women into birth cohorts. Primary data birth cohorts are usually time‐limited for research purposes, and few in Europe were recruiting when the COVID‐19 pandemic began.^74^

Population cohort studies based on existing data sources can have the advantages of being large, population‐based, comprised of exposed and unexposed pregnancies, and both normal and adverse pregnancy outcomes. The quality of such data for studies of COVID‐19 and CA can be judged by several criteria (Table 2)

**TABLE 2:** Criteria for judging the quality of population cohort studies of COVID‐19 and CA with secondary use of existing data sources

Criterion	Explanation
Size of the birth population covered	Large population size is one of the main advantages of using existing data sources, which allows risks relating to rare exposures or specific CA to be addressed.
Quality and completeness of data on CA	Healthcare databases in their “raw” form are operational data that may have poor predictive value or completeness for CA.^75^, ^76^, ^77^, ^78^ A better option is linkage of congenital anomaly registries to healthcare databases with exposure data for all pregnancies. This also allows the inclusion of TOPFA and stillbirths, although TOPFA cannot be linked to prescription data in some countries. Some studies have developed algorithms to improve the predictive value of healthcare data for CA where CA registries are not available.^36^, ^75^, ^76^
Quality of maternal exposure data	The quality of data on COVID‐19 disease symptoms, tests, treatment, or vaccination which are available in electronic healthcare databases.
Quality of data on pregnancy timing and exposure timing	Gestational age at exposure is critical to establish exact weeks of exposure in relation to critical development windows for specific CA. Gestational age at exposure (or pregestational exposure) can be estimated from the date of the prescription or procedure, combined with the expected date of delivery or gestational age and birth date. Primary care databases are particularly prone to very incomplete data on pregnancy timing.^77^
Quality of establishment of study population of mother‐baby dyads	Several countries, for example the Nordic countries and Scotland, have a mother‐baby linkage spine which identifies the healthcare number of the mother for each baby. The most successful linkage uses unique identification (ID) number provided at birth for every individual in the population, but biases may still be present (e.g., preterm babies dying before being allocated an ID number)^79^ and TOPFA may remain unlinked. Studies which conduct linkage based on non‐unique matching variables may have a large proportion of unlinked or incorrectly linked pairs. Mother‐baby dyads need to be present in the study population from at least estimated conception date (or before to allow for periconceptional exposures or preconceptional comparators) to age of baby at confirmation of outcome. When healthcare databases are not population‐based, there may be considerable attrition due to movement during or after pregnancy out of the selected healthcare units, or health insurance schemes, and some such movements of families may be associated with specialist services for children with CA, introducing bias. High proportions of unlinked mother‐baby dyads are a “red flag” for interpretation.

## 4 MULTINATIONAL OR MULTICENTRIC PLATFORMS

For CA, which are relatively rare outcomes, particularly when specific CA are considered, multinational or multicentre collaboration is almost always required for sufficient statistical power. For data sharing, there are a number of infrastructure options (Table 3). For example, the European network EUROmediCAT uses a number of different approaches—a central database for case‐malformed control and signal detection studies, a distributed data approach using a common data model for registries able to link to prescription data for case‐malformed control studies, and a distributed data approach for linkage to population healthcare databases for population cohort studies.

**TABLE 3:** Approaches to data sharing in multinational or multicentre studies

Data Sharing Approach	Advantages and Disadvantages	Analytic considerations	Examples
Central Database, with Common Data Model (CDM)	Useful for rare events such as CA. Since data are already standardised in a common format, data quality improvement processes are performed on an ongoing basis, and collaboration between data providers is established with mutual understanding of data quality, the study can be conducted quickly with appropriate data interpretation. However, the agreement and establishment of an ongoing central database for a network (rather than specific study) is an infrastructure development which takes time and needs a long‐term vision.	When a central database is used, the availability of individual patient data (IPD) from all contributing centres enables complete exploration of the data. Iterative procedures to obtain the best fit for models can be employed. Multi‐level models can be fitted to characterise and adjust for any centre differences both in terms of outcomes, but also in terms of risk factor associations varying between centres. Multiple imputation techniques can be implemented for missing data using the data from all centres.	EUROmediCAT (www.euromedicat.eu) and ECLAMC (www.eclamc.org) have annually updated central databases The US National Birth Defects Prevention Study (www.nbdps.org) and its successor BD steps (www.bdsteps.org) have central databases of IPD, as well as WHO‐TDR registry of pregnancy drug exposure (https://www.who.int/tdr/research/tb_hiv/drug‐safety‐pregnancy/en/) Other networks like ENTIS (www.entis.org), ICBDSR (www.icbdsr.org) and the US NBDPN (www.nbdpn.org) construct central databases of IPD on a study‐specific basis. COVI‐PREG (COVI‐PREG ‐ Département femme‐mère‐enfant ‐ CHUV), a study specific multinational data entry portal.
Distributed Data Network –No CDM or partial CDM	Distributed data models are needed if population electronic healthcare databases or data linkages are used, allowing (IPD) data to remain in the country of origin. A common protocol can be agreed, which is implemented (programmed) by each participating country, who then provides aggregate tables of results and parameter estimates to the co‐ordinating analyst(s). This is quick to set up. The individual patient data (IPD) can be analysed within each centre by experts with knowledge of their own data. However, it is difficult to distinguish real and artefactual differences, or to perform quality control of data or analytic methods; in practice, this model is best performed by collaborative networks who have experience of working together and mutual understanding of data sources. Networks might collate data dictionaries for all participating centres to facilitate protocol development.	When a common protocol is used, but no CDM, results from the different centres can be combined using standard meta‐analytic techniques to obtain overall crude estimates of effect. Each centre may produce adjusted effect estimates which can be combined, but all adjustments will be specific within centre adjustments and hence generalisability is compromised, and any observed differences may arise due to differences in methodology as well as differences in the data. The effect of individual risk factors or confounders cannot be examined across all centres. Data sharing may be limited by small number suppression.	EUROmediCAT studies with population data linkage (www.euromedicat.eu) using EUROCAT CDM for registry data, without CDM for population data. Nordic Collaborations e.g. InPreSS Collaborators ‐ H4P (harvardpreg.org) LIFECYCLE Home ‐ LifeCycle (lifecycle‐project.eu)
Distributed Data Network – Common Data Model, with and without automated data access.	A common data model (CDM) can be constructed, which maps the local data to an agreed framework, and allows the statistical programs to be written centrally rather than by each participating centre. This requires initial investment of time in the construction of the CDM, but allows for greater transparency and standardisation thereafter. The danger is that the centralisation of the script writing also brings with it less involvement of the country‐specific data experts, and active processes to involve them fully are essential. Many types of CDM are now in existence. Multipurpose CDMs such as OMOP require more initial investment as they apply to all data types, and have not been specifically used for the pregnancy situation. The Sentinel CDM has been specifically adapted for pregnancy pharmacovigilance using US databases. Protocol‐driven CDMs such as used by EUROlinkCAT choose a subset of the variables that are needed for the set of studies to be conducted, perform mapping and validation, and subsequently facilitate rapid centralised programming. They can be expanded to new protocols for new studies by adding variables and data sources. Intermediate solutions, such as the CDM of ConcePTION performs syntactic but not semantic harmonisation for pregnancy studies, requiring semantic harmonisation on a study‐specific basis so that in practice building a library of semantic harmonisation algorithms will be required.	Using a CDM with automated data access means that a syntax script from the analysis centre can be sent to all centres and within each centre a model analysing individual patient data will be automatically fitted and different results concerning the relative fits of the model will be sent back to the analysis centre. These results will be automatically collated and a second model for fitting automatically generated and re‐supplied to all centres. This process will continue until the optimum fit across all centres is obtained. Full automation enables complete exploration of the data to occur. An example of this structure being available is the Sentinel System.^80^ Using a CDM without automated data access means that iterative model fitting cannot be used as each syntax script from the analysis centre needs to be downloaded and run individually. Often the capacity for running many models is limited (for example if all output files need to be checked independently for small number suppression ‐disclosure control before being released) and therefore potentially important covariates need to be identified in advance and included in models run by all centres. Risk factor associations can be investigated by performing meta‐ analysis of coefficients in fitted models. This is equivalent to two stage IPD analysis and does not result in dramatic loss of power compared to the one stage method of analysis ^81^.	OMOP OMOP Common Data Model – OHDSI Sentinel Program^82^ www.sentinelinitiative.org ConcePTION www.imi‐conception.eu EUROlinkCAT www.eurolinkcat.eu ^83^ EUROmediCAT (www.euromedicat.eu) case‐control studies link CA registries to prescription data using a CDM and common software.^64^ Vaccine Safety DataLink.^84^

## 5 CHALLENGES AND OPPORTUNITIES

In addressing the CA risks associated with the COVID‐19 pandemic, it is important to maximise use of the data resources we have, while also addressing the major challenges for this area of pregnancy research. CA research should not be an ‘afterthought’ and requires special attention in protocols.

The ability to use existing structures has already facilitated the COVID‐19 response. Examples are obstetric surveillance systems^1^; MothertoBaby^49^ system and repurposing of Zika pregnancy cohort protocols^50^ and Intergrowth protocols^51^ for COVID‐19. Consolidation of pregnancy exposure registries in Europe could be envisaged where national pregnancy cohort portals, run by the Teratogen Information Services or other qualified institutions, collect data on behalf of all the disparate pregnancy registries (including industry registries), thus improving standardisation and comparability of data, providing internal comparator data, and preventing duplication of reporting. The widespread use of apps has created further opportunities for data collection. While direct‐to‐mother approaches for pharmacovigilance and pregnancy cohorts have not yet proved successful^56^ due to challenging issues of trust, confidentiality, data quality and counselling needs of women, apps are a useful adjunct to collect data, in studies where healthcare providers are involved in recruitment and in provision of medical records for CA diagnoses.^43^

**FIGURE 2:**
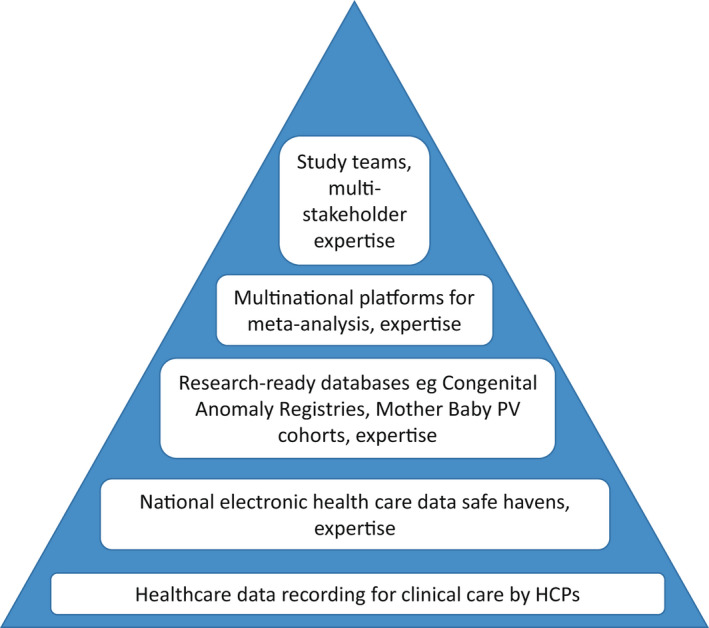
The Data Pyramid for research based on secondary use of data, from healthcare practitioners (HCPs) to study teams

In relation to secondary use of existing data, European countries need to invest in making data more rapidly available, providing safe havens for data access with appropriate data protection, creating ongoing mother‐baby linkage systems (such as in France and Finland; Supplementary Appendix 2), providing for linkage of pregnancies and congenital anomaly registers with population databases of COVID‐19 test results and vaccinations, and providing for linkage of TOPFA to prescription and other healthcare data. Centralising inpatient hospital medication data, which is currently unavailable in most countries,^85^ will be particularly important for COVID‐19, with the use of biologics and injectables which are only delivered in the hospital context. The data flow for secondary use studies can be seen as a pyramid (Figure 2). Balanced investment of resources is needed at every level of this pyramid.

Hybrid approaches to data collection, linking primary and secondary data, are not being used to their full potential in relation to COVID‐19, nor more generally in pharmacovigilance. The most efficient use of research resources would be to concentrate primary data collection on pertinent information not available in existing data sources, and link these data to congenital anomaly registries^86^ and electronic healthcare databases. Hybrid designs could provide internal comparators and reduce the cost and attrition involved in follow‐up. This requires identifiable data and appropriate consent and data protection frameworks to allow such linkage to take place.

We have reviewed four types of data source here, which complement each other in building evidence regarding CA risk. However, there is presently no coordination regarding the identification and testing of signals regarding COVID‐19 related exposures in pregnancy, where the World Health Organisation could play a useful role.

Systematic reviews and meta‐analyses will always be particularly important in gathering evidence for rare outcomes such as CA. However, lack of standardisation and specificity in the reporting of CA, as well as lack of reporting of trimester‐specific exposures, will reduce the potential for meta‐analysis and contribute to delays in obtaining evidence to guide healthcare.

Pregnancy remains a significant area of challenge for COVID‐19 related research. By achieving a fuller understanding of the current problems in the data and systems we have available, and acting to use opportunities and address challenges, we can move to a position where pregnant women can make choices about treatments and vaccines informed by a similar level of evidence as is available to the rest of the population. This is an important part of protecting future generations from the effects of the current and future pandemics.

## Data Availability

Data sharing not applicable to this article as no datasets were generated or analysed during the current study.

## APPENDIX 1 Overview of the main modes of action of medications used or proposed in SARS‐CoV‐2 infections^31^, ^35^

**Table:**

Treatment aim and mode of action		Medication class	Examples
Act against the virus	Direct‐acting antiviral agents	Protease inhibitor prodrug of a nucleoside analog. Nucleosidic analogues	lopinavir remdesivir, favipiravir, molnupinavir
	Passive immunotherapy	antibodies cocktail	Convalescent plasma
	Drugs acting on the way it proceeds at its pulmonary “gate”	monoclonal antibodies acting on the spike protein	Bamlanivimab Casirivimab‐imdevimab
		serine protease inhibitor	Camostat acting on TMPRSS2
		Antimalarial (interfering with glycosylation of the SARS‐CoV‐2 ACE2 Receptor)	Chloroquine, hydroxychloroquine
		Antidepressant (acting on sigma‐1 receptor (SIGMAR1)	Fluvoxamine
Inhibit the cytokine storm	Immunomodulatory therapy anti‐inflammatory drugs/	corticoids	Dexamethasone
		TNF blockers	Infliximab, adalimumab
		IL antagonists	Tocilizumab, sarilumab
		JAK inhibitors	Tofacitinib, baricitinib
Avoid complications	Avoid or cure thrombosis	Anti‐coagulants, Antithrombotic agents	Heparin Aspirin
	Avoid bacterial superinfections	Antibiotics	Azithromycin
	Avoid severe changes in arterial blood pressure	Antihypertensive agents, vasopressor agents	
	Avoid pulmonary hypertension	Phosphodiesterase inhibitor	Sildenafil
Attenuate common symptoms	Fever Cough	Antipyretics Antitussives	Acetaminophen Codeine

## APPENDIX 2 EUROmediCAT data resources for studies of congenital anomaly risk in relation to disease and medication/vaccination exposure^60^

**Table:**

Country	Type of data source
Multicountry ‐ EUROmediCAT Central Database	Currently 21 EUROCAT congenital anomaly registries from 14 countries contribute to the EUROmediCAT Central Database, covering approximately 753,000 annual births. ATC coded medication exposures and maternal disease information. The 21 registries include UK‐Wales, Denmark‐Funen, Italy‐Emilia Romagna and Tuscany, Spain‐Valencian region.
France ‐ EFEMERIS (Évaluation chez la Femme Enceinte des MÉdicaments et de leurs RISques)	This is a database intended specifically to evaluate drug safety in pregnancy, linking available electronic healthcare data and primary care data (for live births) and medical files (for TOPFA). Approximately 10,000 pregnancies per year in Haute‐Garonne area (South West France). Data come from reimboursed drugs, and although there is no EUROCAT CA register, comprehensive data on CA come from child health certificates filled out from birth to 24 months, prenatal diagnosis centres, and hospital data on stillbirths, TOPFA and pregnancy loss.
UK – Wales ‐ SAIL	The Secure Anonymised Information Linkage (SAIL) Databank holds linkable, anonymised individual level data from virtually all healthcare data sources in Wales, with 60,000 annual births, including the CARIS (EUROCAT) congenital anomaly registry https://saildatabank.com/ A similar system in Scotland will soon incorporate a new EUROCAT congenital anomaly register. There are also plans to link the English congenital anomaly registry to prescription data. The CPRD primary care database has CA data of limited quality,^77^ but can eventually be linked to congenital anomaly registry data.
Finland ‐ Drugs and Pregnancy Project	This data infrastructure brings together for 46,000 annual births, data from the Medical Birth Register, Register on Induced Abortions, Register of Congenital Malformations (EUROCAT), Prescription Register and Special Refund Entitlement Register https://thl.fi/en/web/thlfi‐en/research‐and‐expertwork/projects‐and‐programmes/drugs‐and‐pregnancy. Further data, including primary care data, are also available.
Denmark	All national healthcare databases (except primary care) are available at Statistics Denmark, for 61,000 annual births. However, there is no national EUROCAT congenital anomaly registry, only a regional one (Funen County, with 8% of national births).
Sweden	All national healthcare databases (except primary care) are available in Sweden, including a EUROCAT congenital anomaly registry, for 115,000 annual births. No medication exposure data available for TOPFA.
Norway	All national healthcare databases (except primary care) are available in Norway, including a EUROCAT congenital anomaly registry, for 60,000 annual births.
Italy – Emilia Romagna	All the regional healthcare datasources (except primary care) are available, for 35,000 annual births, including a EUROCAT Congenital Anomaly Registry. TOPFA cannot be linked to prescriptions.
Italy ‐ Tuscany	All the regional healthcare datasources (except primary care) are available, for 25,000 annual births, including a EUROCAT Congenital Anomaly Registry. Other new EUROCAT registries which can be linked to regional healthcare data are starting in Milan Metropolitan Area, Mantova, Sicily and Veneto.
Spain – Valencian Region	All the regional healthcare datasources are available for 45,000 annual births, including a EUROCAT Congenital Anomaly Registry. Primary Care records are included, as well as a Vaccine Information System. TOFPA cannot be linked to prescription data. Similar data are available for the Basque Country region.

## APPENDIX 3 Selected resources for Congenital Anomaly studies (for a more extensive list, see Resources Inventory • Global Birth Defects (tghn.org))

**Table:**

**EUROCAT subgroups**, based on ICD9‐BPA and ICD10‐RCPCH codes: (https://eu‐rd‐platform.jrc.ec.europa.eu/sites/default/files/Section%203.3‐%2027_Oct2016.pdf). Well known teratogenic exposures (diabetes, valproic acid, thalidomide, insufficient folic acid) result in specific congenital anomalies, not all congenital anomalies. This is mainly related to the mechanism of action, but different defects may also result from different timings of exposure during organogenesis. Studies that combine all congenital anomalies together may not detect increases in risk of specific anomalies, and it is recommended that as sample size increases, more specific congenital anomaly groups should be analysed. In order to aid meta‐analysis across studies, results should always be disaggregated by specific CA subgroups to as great an extent as possible. EUROCAT subgroups allow this to be done in a standardised manner.
**Minor anomalies for exclusion**. These are frequent anomalies, inconsistently diagnosed, with little medical or functional significance, which can cause considerable “statistical noise” in prevalence rates unless standard exclusions apply. Some are not true congenital anomalies e.g. patent ductus arteriosus in preterm births. Two very similar lists are published by EUROCAT (https://eu‐rd‐platform.jrc.ec.europa.eu/sites/default/files/Section%203.3‐%2027_Oct2016.pdf) and the WHO 9789240015395‐eng.pdf (who.int)
**EUROCAT guidance for calculating Total Prevalence rates** https://eu‐rd‐platform.jrc.ec.europa.eu/sites/default/files/Section%204.1‐%2027_Oct2016.pdf CA cases should include livebirths, fetal deaths from 20 weeks (or a suitable threshold gestational age after which diagnosis is well recorded) and TOPFA (i.e. termination of pregnancy for fetal anomaly after prenatal diagnosis). The denominator for total prevalence rates is all births (live and still). TOPFA can be included in the denominator but are too few in relation to births to make a difference (TOPFA may account for up to 1 for every 100 births in Europe, usually less). Lack of TOPFA in the numerator underestimates the risk of CA. Non‐TOPFA terminations and spontaneous abortions should not be included in either the numerator or the denominator. This is because they are incompletely reported, and incompletely examined for presence of a CA, and being numerous can considerably bias CA prevalence downwards.
**EUROCAT Prevalence rates of major congenital anomalies** https://eu‐rd‐platform.jrc.ec.europa.eu/eurocat/eurocat‐data/prevalence_en Prevalence rates per 10000 births of 92 subgroups per EUROCAT registry, per year, per type of birth. Can be used as external comparator rates, or to help in sample size calculations when planning studies, or to help interpret how unusual the distribution of anomaly types in a case series is.
**ConcePTION Core evidence elements** for generating medication safety evidence for pregnancy using population‐based data contains extensive information on Congenital Anomalies, available at ConcePTION‐D1.2.pdf (imi‐conception.eu)
**CA Registry Data Quality indicators**. Congenital anomaly registries measure the quality of their data, and data quality indicators help to decide which data should be included that meets quality standards. EUROCAT Data Quality Indicators can be found at https://eu‐rd‐platform.jrc.ec.europa.eu/eurocat/data‐collection/data‐quality_en). A set of indicators suitable for registries worldwide has been published by the WHO in collaboration with ICBDSR 9789240015395‐eng.pdf (who.int)
**Pictorial Guides to Congenital Anomalies to aid in identification and coding**. In the high income countries of Europe, health systems generate good quality data on CA, and the challenge for the registry is to access the records (increasingly in electronic form), and to cross‐check between different stages of the baby’s diagnostic journey. In low to middle income countries, the availability of specialist healthcare professionals is much more patchy, and it may be difficult to collect CA data for research and surveillance. The WHO, in collaboration with CDC and ICBDSR, has issued a useful Quick Reference Handbook of selected congenital anomalies with photos and diagrams (9789240015418‐eng.pdf (who.int)). The Global Birth Defects has developed an app to help non‐experts identify birth defects with simple‐to‐use pictorial pathways (https://globalbirthdefects.tghn.org/download‐birth‐defects‐surveillance‐app/). The ECLAMC network in South America have an extensive online database of photos of congenital anomalies Home ‐ Congenital Malformations Browser (atlaseclamc.org)
**Global Birth Defects** website contains an extensive inventory of available resources for Congenital Anomaly Surveillance, Research, Prevention and Care Resources Inventory • Global Birth Defects (tghn.org)
